# Capturing Maize Stand Heterogeneity Across Yield-Stability Zones Using Unmanned Aerial Vehicles (UAV)

**DOI:** 10.3390/s19204446

**Published:** 2019-10-14

**Authors:** Guanyuan Shuai, Rafael A. Martinez-Feria, Jinshui Zhang, Shiming Li, Richard Price, Bruno Basso

**Affiliations:** 1 Department of Earth and Environmental Sciences, Michigan State University, East Lansing, MI 48823, USA; shuaigua@msu.edu (G.S.); mart2225@msu.edu (R.A.M.-F.); lism@ifrit.ac.cn (S.L.); priceri1@msu.edu (R.P.); 2 State Key Laboratory of Earth Surface Processes and Resource Ecology, Beijing Normal University, Beijing 100875, China; zhangjs@bnu.edu.cn; 3 Institute of Forest Resource Information Techniques, Chinese Academy of Forestry, Beijing 100091, China; 4 W.K. Kellogg Biological Station, Michigan State University, Hickory Corners, MI 49060, USA; 5 Great Lakes Bioenergy Research Center, Michigan State University, East Lansing, MI 48824, USA

**Keywords:** UAV, plant detection, distance estimation, field experiments, yield stability

## Abstract

Despite the new equipment capabilities, uneven crop stands are still common occurrences in crop fields, mainly due to spatial heterogeneity in soil conditions, seedling mortality due to herbivore predation and disease, or human error. Non-uniform plant stands may reduce grain yield in crops like maize. Thus, detecting signs of variability in crop stand density early in the season provides critical information for management decisions and crop yield forecasts. Processing techniques applied on images captured by unmanned aerial vehicles (UAVs) has been used successfully to identify crop rows and estimate stand density and, most recently, to estimate plant-to-plant interval distance. Here, we further test and apply an image processing algorithm on UAV images collected from yield-stability zones in a commercial crop field. Our objective was to implement the algorithm to compare variation of plant-spacing intervals to test whether yield differences within these zones are related to differences in crop stand characteristics. Our analysis indicates that the algorithm can be reliably used to estimate plant counts (precision >95% and recall >97%) and plant distance interval (*R^2^* ~0.9 and relative error <10%). Analysis of the collected data indicated that plant spacing variability differences were small among plots with large yield differences, suggesting that it was not a major cause of yield variability across zones with distinct yield history. This analysis provides an example of how plant-detection algorithms can be applied to improve the understanding of patterns of spatial and temporal yield variability.

## 1. Introduction

The adoption of precision agriculture technologies has been increasing in large-scale commercial crop production, largely motivated by the potential advantages in input-use efficiency and cost savings [1]. One such technology is precision planting. Improvements in sensors and agricultural equipment are making it possible to place seeds at an increasingly uniform spacing and depth [2]. The aim is to ensure equal distribution of light, water, and nutrients among crop plants, which is critical for high yields. Inconsistent seed depth can cause uneven timing of seedling emergence, giving early-emerging plants an advantage that often allows them to out-compete neighboring plants [3,4]. Meanwhile, irregular plant distance within the crop row results in plants with more grain in areas of low density [5], but this variation in the growth and yield of individual plants has been shown to be detrimental to the aggregate of the whole stand in key crops such as maize (*Zea mays* L.) [6,7]. A uniform plant stand is also often assumed in crop simulation models for field-level yield estimation [8], and uneven stands can lead to bias and inaccuracy of simulations. Despite the new equipment capabilities, crop stand uniformity can still be affected by spatial heterogeneity in soil conditions, seedling mortality due to herbivore predation and disease, or human error [3]. Therefore, detecting signs of variability in crop stand density early in the season provides critical information for management decisions such as the rates and timing of inputs (e.g., fertilizer, irrigation, and herbicide) as well as for more accurate crop yield forecasts [9]. 

Assessing crop stand uniformity has historically been achieved through crop scouting, that is, the visual inspection of crops. Increasingly, however, spectral, thermal, and LIDAR (Light Detection and Ranging) sensors mounted on unmanned aerial vehicles (UAVs) have been deployed to broaden the extent and frequency of crop scouting missions [10]. In contrast to remote satellite sensing, UAVs can collect measurements even on cloudy days and provide almost real-time information on variables such as crop height and biomass as well as nutrient and moisture levels [11]. These are inferred through their correlation with various vegetation indices, which are algebraic combinations of several spectral bands. Moreover, field variability detected by UAV images can be used to guide variable rate technology systems for precision placement of inputs. In addition to crop density and plant-to-plant distance estimation, UAV applications also include weed mapping and management, crop disease detection, and high-throughput phenotyping in crop breeding [12,13,14,15,16].

Detection of the location of maize plants is a necessary step to remotely assess stand uniformity [16], and spectral data collected with UAV has been used successfully to identify crop rows in a variety of crops [17]. Typically, vegetation indices are calculated and used to isolate pixels with vegetation from the background with simple thresholding techniques or classification algorithms. For example, Jin et al. [18] used the Meyer-Neto vegetation index (MNVI; i.e., difference between the excess green and excess red indices) threshold to identify wheat pixels in RGB images. After image classification, morphology operations are needed to remove some outlier pixels, such as weeds or crop residues with less than a specified number of connected pixels. The next step is to aggregate crop pixels in the binary classification image into contiguous and discontinuous regions with image segmentation methods [18,19]. Crop row detection algorithms are applied to assign crop plants to different rows, often with a Hough transform [20] approach to compute row center points and equations for row lines in the images. The assumption is that all of the crop plants in one row are collinear, and their sinusoids in the Hough space are at the same crossing point, whose coordinates can lead to the searched line. Rovira-Más et al. [21] applied Hough transform in a real-time case study and found the generated crop row lines were in good agreement with a qualitative visual evaluation. Leemans and Destain [22] also successfully localized seed rows under various soil textures and illumination conditions using the Hough transform method. Their results suggested that a prior knowledge of the row spacing increased the detection accuracy [22]. Another common method for row detection is the vanishing point [23], which maps rows using a skeleton of a predefined region. The combination of both methods has been seen to produce good results, with a detection rate of up to 90% in wheat [18].

Recently, Zhang et al. [19] proposed a new method that adjusts the center points of segmentations according to the view angle to calculate maize plant spacing from UAV images at multiple flight heights [19]. This allowed for the identification of maize row and plant distances with a relative error of around 10%, which is sufficient for field-scale research. In this paper we further test the algorithm of Zhang et al. by applying it on UAV images collected from field zones with distinct historical yield levels in a commercial crop field. Our objective was to implement the algorithm to compare the variation of plant-spacing intervals among these zones to test whether yield differences within these zones are related to differences in crop stand characteristics. To our knowledge, this analysis is the first to provide an example of how of plant-detection algorithms can be used to advance our understanding of spatial and temporal patterns of yield variability.

## 2. Materials and Methods

### 2.1. Site Description

The field data was collected from a commercial maize field located in Ionia County, Michigan, USA. This area has a temperate continental climate with warm summers and long, cold winters, with a monthly average daily temperature of 27.8 °C in July and of −1.7 °C in January. Average annual rainfall is 904 mm, normally distributed throughout the year with peak precipitation coinciding with the growing season. The field is 27.6 ha in size, with Dryden and Barry sandy loams as dominant soil types. Following a wheat crop in 2017, the field was tilled with a vertical tillage implement 2 weeks before planting. The corn hybrid G95D32-3220-EZ1 (Syngenta, Switzerland) was planted on May 1, 2018 at a target population of 9.8 plants m^−2^. Plant row spacing was 50.8 cm, with the exception of 60.96 cm between two wheels of the planter, totaling 24 rows over a length of 12.9 m. Nitrogen fertilizer was applied in liquid form at a rate of 62.1 kg N ha^−1^ at planting. The first plants emerged on 9 May 2018.

**Figure 1:**
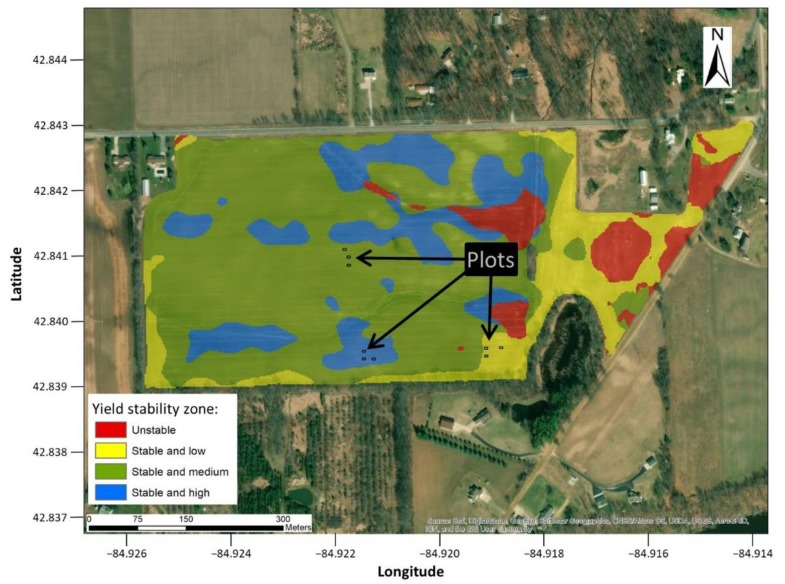
Geographical location of the commercial crop field and the plots established within three yield stability zones.

Yield stability maps are created using historical yield monitor data to improve agronomic management in homogeneous zones and to guide field sampling [24,25,26]. Crop yield, recorded by the harvest monitors as point data at a 2 m interval for each row, was acquired for the field from 2010 to 2018, including 4 years as soybean, 3 years as maize, and 2 years as wheat. The technique of mapping yield stability can be found in ref. [25], which divides the field into four zones: Stable and high yield (SH), stable and medium yield (SM), stable and low yield (SL), and unstable zone (UN). In this study a total of three plots with a size of 5 m in length and 3 m (6 rows) in width were established in each of the stable zones (Figure 1) to test the accuracy of the plant-to-plant distance estimation method.

### 2.2. UAV Flight and Field Data Collection

The Mavic Pro Platinum quadcopter (DJI, China), mounted with a 4K RGB camera, was used to take aerial images. The camera had a 26 mm (35 mm format equivalent) focal length lens and a 1/2.3-inch CMOS sensor to capture images with a size of 4000 pixels × 3000 pixels. The field-of-view angle was 78.8°, which is a key parameter to calculate the spatial resolution of acquired images. The position of the UAV was provided by GPS or GLONASS (Global Navigation Satellite System) and a magnetic compass to maintain the flight direction. All images were collected on 25 May 2018, when most maize plants reached second leaf collar stage and no overlapping was observed for plants with normal row spacing. Cloud cover is often an unavoidable issue in Michigan due to its proximity to the Great Lakes, so some images were taken under sunny skies and others under clouds with light wind. At each plot, we collected images at two flying heights (4 and 5 m), with three replicates for each height. Given these flying heights, spatial resolution of UAV images was 1.1 and 1.4 mm respectively. Camera settings were the same for all of the plots: The shutter speed was set to 1/1000, focal length to *f*2.2, and ISO to 100. From the six images collected at each plot, we selected the best-quality image in regards of minimal blur and vignetting for subsequent image analysis. Since each image only covers one single plot and was processed separately, there was no need to perform ortho-rectification or stitching among images, and distortion effect within one single image can be ignored.

Crop heights of 10 individual plants were measured in each plot, with an average of 10 cm as a global uniform parameter for subsequent image analysis. Plant-to-plant distances were also measured for each pair of adjacent maize plants and were used to validate the estimated results. Each plot was marked by four stakes at corners and a white panel at the center. Non-uniform plant-to-plant distance was observed for selected plots at different degrees. There were also crop residues within the plots, which increased the complexity of crop plant identification. Maize yields were acquired by combined harvester in mid-October.

### 2.3. Image Processing

The method developed by ref. [19] was applied over aerial images to measure plant-to-plant distance. As briefly described in the introduction, there were three steps: (1) Maize pixels are separated from the background using a vegetation index; (2) maize pixels are aggregated into plant objects; and (3) crop rows are calculated based on extracted maize plant objects and thus of plant-to-plant distance for each derived row.

#### 2.3.1. Separation of Maize from Background Using the Excess Green (ExG) Vegetation Index

Since the near infrared band was not contained in the images, the excess green (ExG), a common RGB-based vegetation index, was calculated for green crop extraction [13,27]. This vegetation index is defined according to Equation (1).

(1) $$ExG=\;\frac{2\times g-r-b}{r+g+b},$$

where *r*, *g*, and *b* are the normalized RGB digital number values using the maximum value of each band. Then the ExG images were classified into binary images using a threshold of 0.1, with maize pixels as 1 and background as 0.

#### 2.3.2. Maize Plant Object Extraction and Refinement

Based on the binary image, connected maize pixels were aggregated into maize objects using the image segmentation function in ENVI 5.1 software (Harris Geospatial Solutions, Inc., Boulder, Colorado). Despite the simplicity of maize object extraction, there were many outliers caused by crop residues or weeds growing in the field, which exhibited similar ExG values to maize. Thus, two filters were applied to remove most of these classification errors. As the first filter, two thresholds of object area and shape, defined as the ratio of perimeter to area, were set to remove some small objects. The threshold is defined as 0.3 multiplied by the average value of each parameter of all objects within one image. For example, objects with area less than the pre-defined threshold, 0.3 × A*_object_* will be filtered, where A*_object_* is the average area calculated from the extracted objects. The second filter is designed to merge separated objects of an individual plant into one single object using a morphology operation. This separation occurs when plant leaves or stems are classified as background due to shade caused by sun and view angles. Thus, a dilation operation with a structure of 10 pixels × 10 pixels was applied to the binary images after the first filter and dilated maize objects with overlap were merged into one maize object. The structure size was determined by the spatial resolution of images and estimated intervals among separated objects. The final binary image was used for the subsequent processes.

#### 2.3.3. Plant Row Detection and Plant-to-Plant Distance Calculation

First, the centroid of the maize object is considered to be the location where the planter placed seeds, so the plant-to-plant distance is the interval among object centroids. In order to determine crop rows, the central point of each maize object on the binary images was adjusted to its real position in the field based on the 3D spatial relationships between camera, plant object, and plant height. This modification accounts for the displacement of maize plants away from the nadir point caused by optical distortion. Principles and a schematic detailing the process of locating the position of maize plant are provided by ref. [28]. After the adjustment of the location of centroids, a random centroid was selected to generate an initial line to cross this point based on a predetermined crop row angle, which was 90° in this experiment. Then a buffer area, with a distance of *w*/2 (*w* is the crop row space, 50 cm here), was created around this line to include maize objects within this region. The average position of all included centroids was calculated, and the initial line was moved to cross this averaged point, which was determined as the final crop row. This process was repeated until every centroid was assigned to its specific row. Finally, all the centroids within one row were perpendicularly projected to the crop row line based on the minimum distance criteria, and the plant-to-plant distance was calculated as the length between two adjacent projected centroids.

### 2.4. Performance Evaluation

Performance evaluation was conducted to assess the accuracy of both maize object identification and within-row spacing. For maize object identification, plant number within each plot was counted from the classified binary image, which was compared to the digital count of maize plants directly by visually interpreting images. We calculated precision (i.e., the fraction of identified features that were indeed maize plants) and recall (i.e., the fraction of all maize plants that were correctly identified) using the following equations:

(2) $$Precision=\frac{TP}{TP+FP},$$

(3) $$Recall=\frac{TP}{TP+FN},$$

where *TP*, *FP*, and *FN* indicate the numbers of true positives, false positives, and false negatives.

An agreement index, *d_e_*, was used to validate estimated maize interval distance using Equation (4):

(4) $$d_{e}=\frac{\sum_{i=1}^{n}\left|d_{ie}-d_{im}\right|}{n},$$

where *d_ie_* and *d_im_* indicate the estimated and measured within-row crop distance. In this study the estimated distances based on FP and FN were not considered in the assessment. For example, if one maize plant was not detected, then the estimated distance between its adjacent plants was discarded. Thus, *n* indicates the number of correctly classified maize plants. In addition, the ratio of *d_e_* to the measured distance, *r*, was calculated to quantify the relative error.

### 2.5. Test of Hypotheses

Plot-level measurements of maize yield, stand density, plant distance mean, and standard deviation were subjected to one-way analysis of variance (ANOVA), with the null hypothesis of equal means across the three yield stability zones (SH, SM, and SL). If the test indicated a significant effect of yield-stability zone, then we conducted mean separation procedure with the Tukey adjustment. We also fitted simple linear regression analysis to test the relationship of stand density to plant distance mean and standard deviation. All tests of hypothesis were conducted with a significance level α = 0.05 using the R statistical software (version 3.5.2) [29], augmented with the packages *emmeans* [30] for mean separation and *dplyr* [31] and *ggplot2* [32] for data manipulation and visualization, respectively.

## 3. Results

### 3.1. Accuracy of Maize Plant Identification

**Figure 2:**
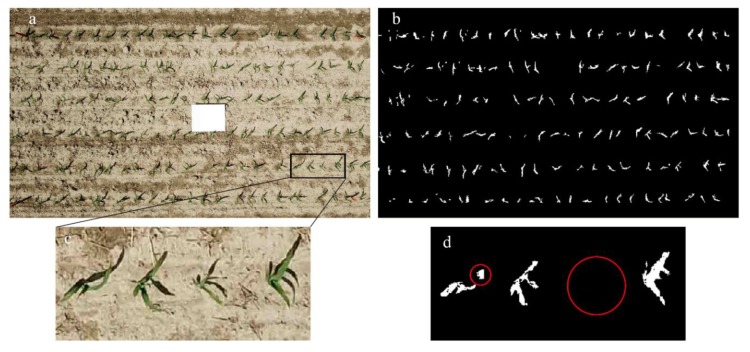
Example of maize plant identification and plant distance calculation (**a**) and (**b**), and associated classification errors (**c**) and (**d**) with red circles in (**d**).

The accuracy of maize identification for all plots is shown in Table 1, and Figure 2 shows the UAV image and associated extracted maize plants in the third SH plot. All precision values were higher than 95%, indicating that less than 5% of maize plants were not identified. This may occur due to inappropriate thresholds on the ExG image or the two filters. For example, in Figure 2c,d, one maize plant was misclassified as background because its small size is less than the threshold setting in the first filter. Another case was highlighted in the left red circle, in which the fragmented segments of one maize plant were not merged due to a larger distance than threshold settings in the second filter. Recall values were 100% for most plots and 97% for the third SH plot and 99% for the second SM plot (Table 1). These FN cases are caused by the similar ExG values of weeds and shade on the maize, which often occurs during the field flight. Overall, these results highlighted the ability of UAV-based maize plant identification at the plot scale.

### 3.2. Performance of Maize Plant Distance Estimation

**Figure 3:**
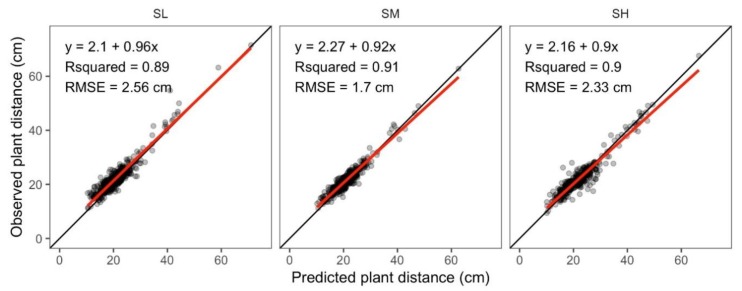
Estimated vs. measured plant-to-plant distance in three yield stability zones.

Observed plant-to-plant distances ranged from 9.4 to 71.5 cm (Figure 3). There were 18 (5%), 8 (2%), and 14 (4%) measurements in the SH, SM, and SL plots larger than 40 cm. Less than 1% of maize distances were also smaller than 12 cm. These small and large intervals were associated with extra and missing plants, which increased the spatial variation of crop yield in uniform stands.

Figure 3 shows that all the coefficients of determination (*R^2^*) between measured and estimated plant distance were around 0.90, and the regression lines of SH and SM were parallel to the 1:1 line with intercepts close to 0. The averaged *d_e_* was 1.7 cm for SH plots, 1.4 cm for SM plots, and 2.0 cm for SL plots, resulting in 75 percent of relative errors that were less than 10%. The slightly large error in the SL plots resulted mainly from uneven emergence rather than the assumed uniform crop height in the algorithm, which in turn affected the calculation of maize centroid locations. The global ExG threshold also introduced some uncertainties in characterizing maize plants; for example, some parts of leaves were classified as background (Figure 2c,d). This also caused inaccurate estimations about maize centroids. Nevertheless, we considered the estimation acceptable for testing our hypothesis.

### 3.3. Plant-to-Plant Distance Across Historical Yield Zones

**Figure 4:**
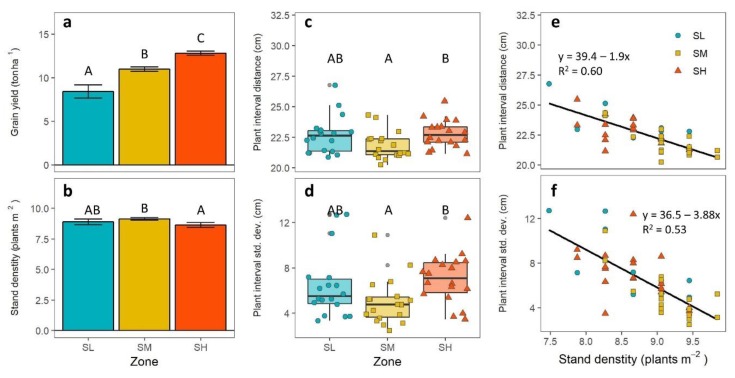
Grain yield (**a**) and plant stand characteristics (**b**–**d**) across plots in zones with stable-low (SL), stable-medium (SM), and stable-high (SH) yields. Error bars in columns indicate standard error of the mean. Treatments with same uppercase letters indicate that means are not significantly different to a Tukey mean comparison test (*p* < 0.05). Fitted linear relationship of stand density and mean plant interval distance (**e**) and standard deviation (**f**).

SH plots produced the highest yield with an average of 12.8 tons ha^−1^, while average grain yield was 11.0 and 8.4 tons ha^−1^ for SM and SL, respectively (Figure 4a). Significant differences (*p* < 0.001) were found among three groups of maize yield, which is consistent with the theory that historical yield stability maps can be used to identify zones with distinct crop yield level [25]. Plant density, which was derived from the predicted maize plant count data, was significantly different across yield stability treatments (*p* = 0.043), averaging 8.6, 9.1, and 8.9 in the SH, SM, and SL plots respectively. Only the means between SH and SM were statistically distinct (Figure 4b).

The average and standard deviation of plant-to-plant distance also differed significantly across zones (*p* = 0.032 and *p* = 0.042, respectively; Figure 4c,d). Stable-high plots had the greatest average plant distance (22.8 cm) and standard deviation (7.1 cm), whereas SM had the smallest average plant distance (21.8 cm) and standard deviation (5.0 cm). Plant density was negatively associated to both average plant distance (*p* < 0.001; *R^2^* = 0.6) and standard deviation (*p* < 0.001; *R^2^* = 0.53): Plant density and standard deviation decreased as plant density increased (Figure 4e,f).

At the plot level, however, final yield was not found to be associated with plant density (*p* = 0.28), average plant distance (*p* = 0.95), or plant distance standard deviation (*p* = 0.63), which suggests that other factors besides spatial variations of maize plant intervals may be the cause of the lower yields in SL and SM. This result indicates that the small differences observed in plant spacing non-uniformity were probably not an important factor in determining crop yield under this environment.

## 4. Discussion

Although modern planters are designed to place maize seeds uniformly in the furrow, uneven spacing among plants is common in practice. Though negative effects of plant spacing variability and uneven emergence have been documented in the literature, the nature and magnitude of the effect have been a matter of some debate. For instance, Liu et al. [33] only found weak correlations among plant growth, maize grain yield, and plant spacing variability by using six plant spacing treatments with standard deviations from 6.7 to 16.2 cm [33]. Lauer and Ranking [2] also did not observe significant effects of plant spacing variability on maize plant lodging or yield. However, another study [7] that assessed the impacts of planter type, speed, and tillage on stand uniformity and yield in Ontario with conventional tillage and no-tillage systems showed a reduction of yield of 35.9 kg ha^−1^ of grain for each centimeter increase in within-row plant spacing variability, while yield reduction increased to 293 kg ha^−1^ per day of delayed emergence [7]. Besides these conflicting findings, it is important to note that these experiments were conducted with defined standard deviations in plant distance, which is more heterogeneous at the field scale. Additionally, even when plant spacing variability does lead to extreme plant-to-plant yield variability, the influence on stand yield have been found to be localized, generally to less than 0.5 m within the row [34].

Our results showed that plant spacing variability differences were small over plots with significant yield differences. In fact, the less uniform stands occurred in the plots with the highest yields (Figure 4), contrary to what we would expect if yields were influenced by heterogeneous stands. This suggests that plant stand differences in our measurements were not a major cause of yield variability across zones with distinct yield history. Yield gaps were most likely due to other factors, such as differences in soil nitrogen and water availability or topographic position [25]. Given that uneven plant distance is almost unavoidable in real-world situations, more experiments are still needed to understand the interactions of plant spacing variability, crop growth, and yield under a variety of soil, weather, and management practices.

Unmanned aerial vehicles provided reliable images for mapping maize plants in this study. All precision values were higher than 95%, and most recall values were 100%, which is consistent with other similar studies [16,19]. This suggest that the method proposed could be an efficient tool for scientists to perform plot-level experiments in further investigations, such as precision crop phenotyping research [16]. Yet, important challenges remain for the operational field-scale deployment of UAVs in agricultural settings, especially in terms of reproducibility. Previous work by Svensgaard et al. [35] comparing the spectral measurements of cereal genotypes by varying cameras, light conditions, and flight altitudes, found that reproducibility was mostly affected by cameras, while no significant impacts were observed for light conditions and flight altitudes [35]. Clearly, increasing flight altitude would also be needed to reduce flight times for whole-field applications. Zhang et al. [19] showed that our maize plant identification algorithm works well up to 5 m flight height, except for when plant distances are very small (9 cm). Yet, at heights greater than 5 m the accuracy of maize plant identification would likely decrease due to increased mixed crop leaves and soil background spectra, affecting the calculation of the centroid of plants. This problem can be potentially fixed in whole-field applications by increasing the resolution of images captured to reduce spectral mixtures, but this could be at the expense of greater computation costs and data storage requirements (i.e., greater number of pixels) compared to lower resolution images. Therefore, investigating approaches to reliably identify plants at much higher altitudes (e.g., 10–20 m) is needed.

Upscaling to real operational monitoring would also require additional image processing such as radiometric calibration, ortho-rectification, and stitching. Fixed thresholds on the ExG and area and shape parameters also may not perfectly delineate the shape of all maize plants, as fragmented segments, incomplete leaves and plant overlapping can introduce error. Similarly, the presence of weeds with similar spectra to maize plants can introduce error in the classification. While weeds outside the row can be easily distinguished from target crops using thresholds on their distances to the nearest row, identification and elimination of in-row weeds is prerequisite to maintain the accuracy of plant interval distance calculation [36]. An adapted local thresholding technique may be useful for dealing with spatial variation of crop growth or in-row weeds. More advanced methods including deep learning can also be used for this purpose [37]. Finally, here crop height was set as uniform in our analysis to reduce the impacts of uneven emergence on the plant distance estimation, which created errors during the projection of plant centroids from image to field domains. These errors would most likely increase when upscaling to the field in operational applications due to larger variations in crop growth occurring at the field scale. Thus, future work should evaluate the integration of LIDAR point cloud data with multispectral images to estimate plant-level crop height to improve the identification of maize plants and distance calculation.

## 5. Conclusions

We demonstrated the potential of using UAV images for monitoring maize stand count during early vegetative stage, which could be especially helpful for guiding in-season management decisions. The poor relationship between crop yield and plant interval distance was mainly due to similar plant interval non-uniformity across various yield zones, while yields differed significantly across zones. This observation indicated that other factors such as soil or topography might have greater influences on final crop yield than crop spacing non-uniformity under this environment. Thus, determining the dominant factors in crop yield variability requires more knowledge under various environments and management. As for the accuracy of the maize plant detection method, the common missing or extra plants were successfully recognized with precision and recall accuracies greater than 95% for all plots. There was a good consistency between calculated and ground measured plant-to-plant distance with all *R^2^* around 0.9 and average *d_e_* < 2.0 cm, highlighting the ability of our method to detect plant interval at the plot-scale and its potential use in agronomic research. Nevertheless, issues such as in-row weeds identification, the mixing of crop leaves and soil background spectra at higher flight altitudes (>10 m) and variable plant heights, would need to be resolved before upscaling our approach to the whole field for reliable performance in operational applications.

## Figures and Tables

**Table 1:** Results of maize plant identification with its associated accuracy.

Stability	Plot	# of Plants	TP	FP	FN	Precision (%)	Recall (%)
SH	1	131	126	5	0	96	100
SH	2	129	125	4	0	97	100
SH	3	135	131	4	4	97	97
SM	1	138	136	2	0	99	100
SM	2	141	135	6	1	96	99
SM	3	139	134	5	0	96	100
SL	1	136	133	3	0	98	100
SL	2	132	129	4	0	97	100
SL	3	139	136	3	0	98	100
